# Fracture Incidence of New Reciprocating Nickel–Titanium (NiTi) Files: A Cross-Sectional Retrospective Study

**DOI:** 10.7759/cureus.67762

**Published:** 2024-08-25

**Authors:** Youssef Algarni

**Affiliations:** 1 Endodontics, King Khalid University, Abha, SAU

**Keywords:** premium blue files, tiadent, root canal, root, fracture, reciprocation, rotary, files, endodontic

## Abstract

Aim

This study aimed to evaluate the frequency of fractures in a new single-file reciprocating system used by endodontists.

Methods

Reciprocal systems were used to evaluate endodontist-treated patients' periapical radiographs and dental records. For the study, the kind of tooth, the quantity of root canals completed, the quantity of broken instruments, and the fragment's size were all recorded. A fracture risk calculation was made for every kind of tooth, root canal, and root third. The new reciprocating systems' fracture incidence was compared at a 5% significant level using the chi-squared test varied between 2 and 6 mm.

Results

There were a total of 5,066 root canals (2,128 teeth) from 1,818 patients. In single-file reciprocating systems, the incidence of fractured files relative to the number of instrumented canals was 22 (0.43%). The mesiobuccal root canal of mandibular molars and maxillary molars had the greatest breakage rates, which were 7 (36%) and 6 (27%), respectively.

Conclusion

The incidence of instrument fractures was lowered when linked to the usage of the Premium Blue reciprocating system in endodontic therapy.

## Introduction

Despite their high degree of flexibility, automated endodontic files have the potential to break during the root canal preparation process [1-2]. This could have an impact on the root canal treatment's outcome [3]. Numerous factors, including the operator's experience, design of the instrument and manufacturing process, technique of instrumentation, and canal geometry, are involved in file fractures [4,2,5].

Concerning clinical trials of nonclinical and implant safety, first-generation reciprocating equipment has been reported to present low rates of fractures. An incidence of Cunha et al. [6] described that 13% of WaveOne devices utilized in one clinical instance had crust formation in the samples examined. Crust formation rates observed for WaveOne preclinical use at differing time points are summarized in the article. In contrast to that, Bueno et al. [7] reported that the instruments WaveOne and Reciproc introducing up to three cases had an incidence of 0. 26%. Shen et al. in their study found out that the value of *r* was 0. With single-use WaveOne coating, the incidence rate was 5%, and there was not even a slight fluctuation in the fracture rates between dentists with fewer and more clinical hours. Students within the endodontics graduate program who used Reciproc and WaveOne instruments several times with different patients reported an incidence of 0. In another study, the quality of life (QOL) reported was 92% according to Caballero-Flores et al. [8]. They discouraged the use of instruments in six randomized controlled trials (RCTs), pleading that the latter was most likely to fracture with continuous and frequent usage.

The reciprocating file system known as the Premium Blue is a new generation of endodontic instruments intended for flexibility with durability. The files are coated with something in order to make them have a longer life span and be more resistant to corrosion. The presented system is envisaged to decrease the number of file fractures and enhance the general outcomes of endodontic procedures. Before the clinical study to be described here, the manufactured Premium Blue system went through several in vitro tests to determine the mechanical characteristics of the material including flexibility and cyclic fatigue endurance. These studies were intended to mimic the stresses that are experienced in root canal procedures, and in all these studies, the performance of this system was reported to be better than that of the previous models of the rotary system. In vitro observation of the Premium Blue files reflected better resistance toward file fracture and better suitability in curved canals.

When instrumentation is carried out by skilled endodontists, the incidence of fractures in a single-file reciprocating system has been shown in several clinical trials [6,7,8,9]. Shen et al. [10] compared graduate-level endodontists with experienced endodontists, finding that both groups had a low fracture risk when WaveOne files were utilized. This study aimed to evaluate the frequency of fractures of single-file reciprocating devices used by endodontists in clinical root canal treatments.

## Materials and methods

Radiographs and dental records of patients seen for 13 months by a single endodontist were used in this cross-sectional retrospective analysis. The study was conducted in the Department of Endodontics of a dental institute. The ethical committee has approved conducting this study, with reference number 00251. The study included patients who underwent root canal treatment with reciprocating systems, provided informed consent for access to their dental records, and included both initial root canal treatments and retreatment cases for a comprehensive analysis of fracture incidence in various clinical scenarios. Exclusion criteria included patients who did not consent, incomplete dental records or radiographs, and cases using non-reciprocating systems.

TiaDent, USA's Premium Blue reciprocating file system, was used in this study. A total of 2,128 root canal procedures were carried out on 1,818 patients, resulting in the instrumentation of 5,066 root canals on 2,128 teeth. The file system utilized in this study was TiaDent, USA’s Premium Blue reciprocating file system, designed particularly for single-file root canal preparation. Three types of primary teeth were used in the study, and they included the following: anterior teeth, the others being the lower and upper anterior teeth. Therefore, the upper and lower premolars have a common name, which is premolars. "Molar" is used to refer to both the upper and lower molars. The file dimensions were T20, T25, T40, and T50 formats of file sizes, which are also available in the Premium Blue system. Accordingly, the fracture rates and the performance of their lumens were assessed at these sizes. The apical and middle thirds of the root canals, which are located within the tooth, were the focus of the analysis.

The approach comprised multiple crucial phases to guarantee precision and uniformity in the process. In order to reduce the danger of canal blockage, the glide route had to be established and the root canals had to be navigated using a size 10 K-file. After that, debris was removed from the coronal section of the root canal by cleaning it with a 5.25% sodium hypochlorite solution. The canal was instrumented using the Premium Blue reciprocating files, which were used to clean and shape the canal using three pecking strokes. The working length of the apical part of the canal was found to be 1 mm coronal to the foramen, and it was similarly prepared and irrigated. All the files were used only in one patient to ensure that they performed optimally and none were interchanged between patients. The purpose of this methodical approach was to provide a definitive assessment of the effectiveness of file fracture and the rate of fracture and how it varied according to specific kinds of teeth.

One file shaping is intended for Premium Blue instruments. Thus, one reciprocating device is sufficient to prepare a root canal entirely. It comes in three different sizes, i.e., T25, T40, and T50, each with a unique taper and tip size. T25 prepares the root canal to 0.25 mm in diameter, tapering it throughout the first apical millimeters to a diameter of 0.08. T40 prepares the root canal to 0.40 mm in diameter, tapering it throughout the first apical millimeters to a diameter of 0.06. T50 prepares the root canal to a 0.50 mm diameter, tapering it throughout the first apical millimeters to a 0.05 taper. T20 prepares the root canal to 0.20 mm in diameter, with the first apical millimeters having a 0.05 taper.

The single-file system is designed for single-file root canal preparation, suitable for simplified cases. It is effective in moderately curved canals due to its flexibility. It is suitable for general endodontic procedures in various tooth types. However, it may not be ideal for complex or highly curved canals where multiple files or systems are needed. It is also suitable for cases with severe canal calcifications that may impede the instrument's progress.

Therapeutic or prosthetic goals were recommended for all the patients who needed root canal treatments. It was found that a minimum of 643 participants would be required based on another study conducted by Cunha et al. [6], where they noted an overall fracture prevalence of 0.42% of the teeth treated; assuming that the true distribution of the entire population is normally distributed, the error range would be approximately 0.50% and the type I error would be in the 0.05 level [11]. Consequently, they found 750 teeth overview sufficient.

Each case is filed with a size 10 K-file to establish the initial glide path and to negotiate the root canals, by proceeding along the intended plane to the orifices. The coronal section of the root canal was next prepped by rinsing it with 5.25% sodium hypochlorite. Apart from the use of ProTaper with a reciprocating motion, the canals considered were enlarged for approximation, and the coronal section of the root canal was rinsed with 5.25% sodium hypochlorite solution after they use a reciprocating file, complete with three pecking strokes. As similarly done in the middle third, the apical third was also prepared by the prior stated technique, and then the working length was determined at 1 mm coronal to the foramen.

According to the single-use policy, each file was only used once. The type of tooth, the quantity of root canal treatments, and the quantity of fractured files were all calculated for an incidence study. Using the method described by Pruett et al. [12], the periapical radiographs of the root canals containing broken instruments were analyzed to determine the size, location, and radius of the fragment, as well as the root canal's angle of curvature. Based on root canal, root thirds, and tooth type, the fracture risk was computed.

However, the data collection was cross-sectional and the data analysis was performed by one researcher, so there is likely to be some reliability and potential observation biases. However, due to the methodological limitations of the research and the need to improve data accuracy, the following measures were taken. The observer had a code of procedure, which involved measurements of outcome data and analysis of the results to avoid the researcher’s influence. All results were reviewed and discussed with another analyst to ensure validity was attained.

**Figure 1:**
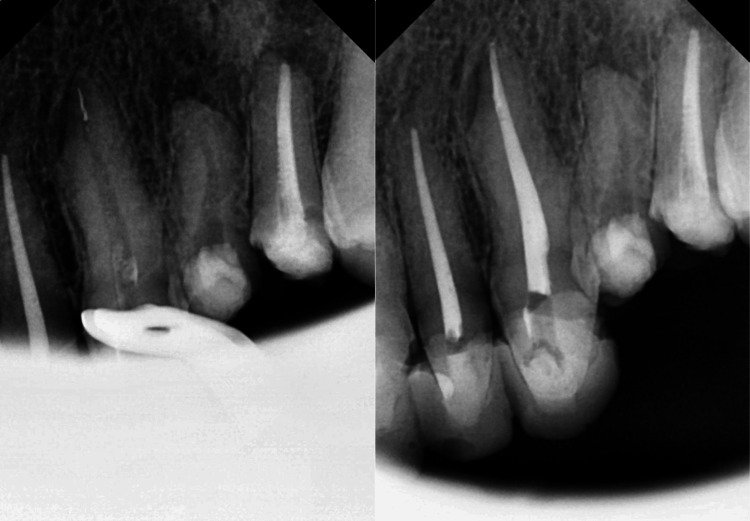
Left side: fractured file in the apical region of the upper premolar. Right side: obturated canal.

Statistical analysis

Chi-square tests were used in the statistical analysis to assess association with variables, and descriptive statistics were used to determine the fracture occurrences for various tooth types, root canal locations, and instrument sizes.

## Results

Three primary tooth types were identified in the study based on the root canal preparations: front teeth, premolars, and molars. There were 311 upper and 214 lower anterior teeth, totaling 525 anterior teeth. There were 289 upper and 311 lower premolars among the 600 teeth that were classified as premolars. There were 1,003 total teeth, 514 upper and 489 lower molars, making up the majority of the teeth. The findings showed that the mesiobuccal canals of mandibular and maxillary molars, in particular, showed the highest frequency of file fractures. On the other hand, the lowest breakage rates were found in the anterior teeth.

In 13 months, 2,128 root canal procedures were carried out on 1,818 patients, resulting in the instrumentation of 5,066 root canals on 2,128 teeth. One-file reciprocating methods were used to instrument 525 anterior teeth, 600 premolars, and 1,003 molars among these teeth. No instruments were thrown out throughout the study period because the handle had changed or the plastic had deformed, and there were no discernible changes between the two study periods (X2 = 0.95, P = 0.3297). Twenty-two (0.43%) of the files fractured overall, with the total number of root canals. The mesiobuccal canal of the maxillary molars had a fracture rate of 6 (27%), whereas the mesiobuccal canal of the mandibular molars had the highest incidence of 8 (36%). In terms of where the fragment was located inside the root canal, the apical third saw 18 (81%) fractures, whereas the intermediate third saw four (19%). The shattered segments varied in length from 2 to 6 mm.

A total of 2,189 Premium Blue instrument files were used without fracture failure, resulting in the instrumentation of 5,044 root canals. Despite this, fractures did occur in 22 (0.43%) root canals and 22 (1.03%) teeth. When considering the different sizes of Premium Blue instruments, the T25 and T20 files had the highest fracture incidence, with 11 (50%) and 10 (45.45%) separations, respectively, accounting for 95% of the fractures. The T40 file had only one (4.45%) fracture, and the T50 file had none, as shown in Figure 1.

**Figure 2:**
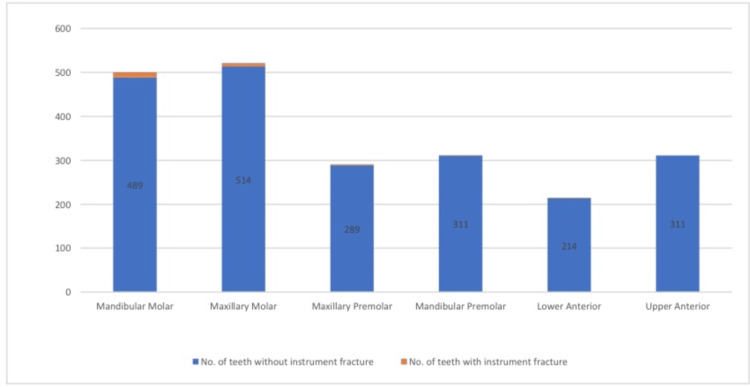
Frequency distribution according to the instrument fracture from a single-file reciprocating system that occurred in the tooth

For the distribution of fracture files among the different sizes of Premium Blue instrument files, the fracture incidence was the greatest for the T25 and T20 files (95%), followed by the T40 file (17%), and no fracture was detected in the T50 file. The distribution of fractures according to the tooth type showed that molars experienced the highest number of fractures, with seven (1.36%) fractures in the upper molars and 11 (2.25%) in the lower molars. By contrast, anterior teeth had the lowest incidence of fractures, with no fractures in the upper anterior teeth and only one (0.47) fracture in the lower anterior teeth.

Specifically, the fracture incidence was 0 (0.00%) for the upper anterior teeth, one (0.47%) for the lower anterior teeth, two (0.69%) for the upper premolars, one (0.32%) for the lower premolars, seven (1.36%) for the upper molars, and 11 (2.25%) for the lower molars. Figure 2 shows the percentage distribution according to the instrument fracture from a single-file reciprocating system that occurred in the tooth.

**Figure 3:**
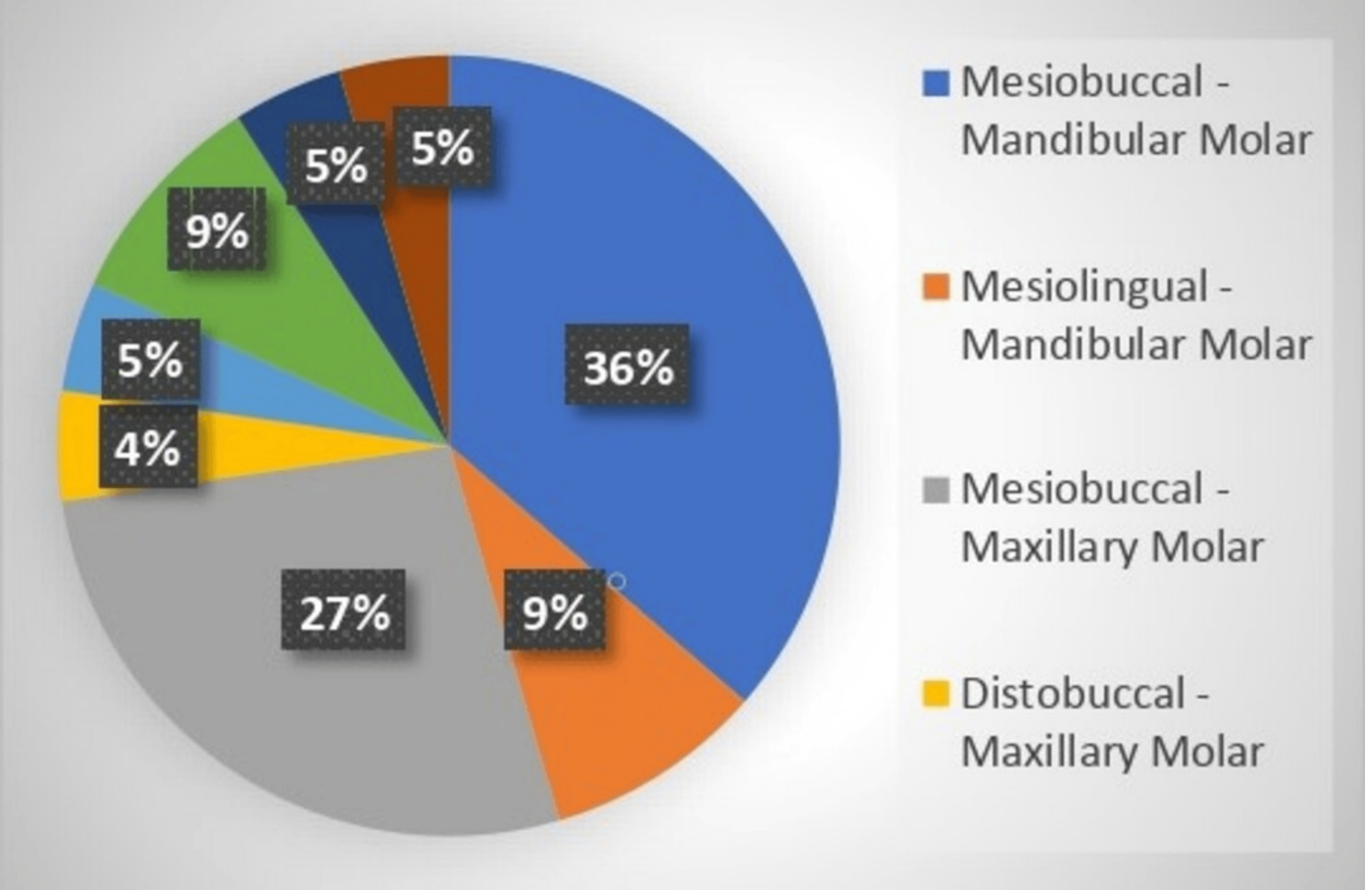
Percentage distribution according to the instrument fracture from a single-file reciprocating system that occurred in the tooth.

Table 1 shows that the fracture incidence of the files in relation to the number of root canals was 0.43% and fractures occurred in 22 (0.43%) root canals and 22 (1.03%) teeth. Eighteen (81%) of the fractures occurred in the apical third of the root canal, and four (19%) of the fractures occurred in the middle third of the root canal. The T25 and T20 files had the highest fracture incidence, with 10 (45.45%) and 11 (50%) separations, respectively, making up 95% of the fractures. The T40 file had only one fracture (4.55%), and the T50 file had no fractures.

**Table 1:** Percentage distribution of Premium Blue instrument fracture among the sizes

File size	Number of teeth (N = 2128)	Number of root canals (N = 5066)	Number of separations	% fracture (teeth)	% fracture (canals)	% of total fractures
T20	2128	5066	10	0.46	0.19	0.43%
T25			11	0.51	0.21	0.43%
T40			1	0.04	0.01	0.43%
T50			0	0.00	0.00	0.43%
P Value				0.032	0.020	
α^2^ value				1.901	1.867	

Table 2 shows that the greatest incidence of fractures was in the mesiobuccal canal of the mandibular molars (7 (36%)), followed by the mesiobuccal canal of the maxillary molars (6 (27%)). The molar teeth had the highest number of fractures, with seven (1.36%) in the upper molars and 11 (2.25%) in lower molars. The anterior teeth had the lowest incidence of fractures, with no fractures in the upper anterior teeth and only one (0.47%) fracture in the lower anterior teeth.

**Table 2:** Percentage distribution of fracture associated with the type of teeth

Tooth type	Upper anterior	Lower anterior	Upper premolar	Lower premolar	Upper molar	Lower molar	Total	P-value (X^2^)
Number of teeth	311	214	289	311	514	489	2128	
Number of separations	0	1	2	1	7	11	22	
% fracture (teeth)	0.00%	0.47%	0.69%	0.32%	1.36%	2.25%	1.03%	0.013 (1.370)
% fracture (canals)	0.00%	0.02%	0.04%	0.02%	0.14%	0.22%	0.43%	0.147 (0.685)

The study found a low overall fracture incidence of 22 (0.43%)for root canals and 22 (1.03%) for teeth, with the highest fracture rates observed in the molar teeth, particularly in the mesiobuccal canals of the mandibular and maxillary molars. The majority of fractures occurred in the apical third of the root canals, and the most affected file sizes were T20 and T25.

## Discussion

In root canal procedures, instrument separation is frequently not only an accident; rather, it can be caused by a number of important variables. Severe curvatures or calcifications in the canal might put an excessive amount of stress on the files, raising the possibility of separation. Furthermore, even though NiTi files are made to be strong and flexible, their intrinsic qualities might nevertheless result in fractures under difficult situations. Another important factor in file breakage is operator technique; inappropriate motion, excessive force, or poor handling can all lead to file breakage. Misuse or overuse of the file can lead to separation even in the case of single-use policies. Even though they are uncommon, manufacturing flaws might cause an instrument to fail before its time. Last but not least, insufficient canal preparation, such as not creating a suitable glide path or doing insufficient cleaning and shaping, can increase stress.

As the root canal treatment performed by endodontists, single-file reciprocating systems have a fracture rate of 0.13% to 0.21% when used just once and up to 0.26% when reused [6,7]. The complexity of root canal treatment referred to an endodontist may account for the highest fracture incidence in the current study. To increase their resistance to fracture, endodontic instruments' alloy, design, and instrumentation methods have all been altered [12-15]. However, inadvertent automated Ni-Ti instrument fractures may occur during the root canal preparation procedure [16-17,6-9]. The current cross-sectional retrospective analysis has shown that a novel single-file reciprocating system has fewer broken instruments when used by an experienced endodontist following a single-use policy.

In mandibular molars, the apical part of the mesiobuccal root canal showed the highest incidence of fractures. According to Sattapan et al. [1], this result is consistent with other studies [6,9]. This was expected due to the anatomical complexity of such root canals, which have double curvatures that are not always visible radiographically. The fragment's most common size was 5 mm, which is consistent with findings from earlier research [1,18,8], which show that Ni-Ti instrument fractures tend to happen closer to the instrument's tip.

Although these investigations are crucial in identifying the variables that may affect this resistance, more reliable interpretations have come from clinical observations. The current analysis did not standardize parameters like tooth type, angle and radius curvature, length, and root canal diameter to estimate the frequency of fracture single-file reciprocating systems in a clinical scenario. According to laboratory research, reciprocating motion resists fatigue better than continuous rotating motion [13,15]. The findings of Tzane-takis et al. [19] and Iqbal et al. [20], who reported fracture incidences of 0.82% and 0.67% for continuous rotary systems, respectively, supported the results of the current study, which revealed a fracture incidence of 1.03% for single-file reciprocating systems. This demonstrates how the outcomes of in vivo and laboratory research might differ.

Based on concerns regarding cross-infection and file damage during root canal preparation, the manufacturer advises against reusing reciprocating files. The Department of Health of the United Kingdom (2007) highlighted the risk of variant Creutzfeldt-Jakob disease (vCJD) transmission from reused endodontic files as the primary reason for potential cross-infection [21]. The actual danger of prion transmission to patients during endodontic treatment is incredibly minimal, despite the theoretical possibility [22]. The American Association of Endodontics and the Canadian Academy of Endodontics special committee found that the single-use requirement for endodontic instruments was not warranted due to the danger of vCJD [23]. The Australian and New Zealand Academy of Endodontists likewise backed this opinion by offering factual evidence that disproved the theory that endodontic file reuse could lead to cross-infection [24].

Additional data suggest that instruments used in reciprocating motion have a longer lifespan than those employed in continuous rotation [22]. According to studies, single-file reciprocating systems may instrument up to nine root canals without encountering any problems. To guarantee the precision and integrity of the findings, each tooth received root canal therapy using brand-new, sterile files for this study. Several studies have examined the relationship between clinician experience and the incidence of instrument breakage. It was found that both novice and experienced endodontists had a low incidence of fractures when using WaveOne files, indicating that the experience level did not significantly impact the fracture rate with this system [25].

Compared to the total root canals, 22 (0.43%) of the files fractured overall. The maxillary molars had the lowest breakage rate (27%), while the mandibular molars had the greatest frequency (36%), in the mesiobuccal canal. Eighty-one percent (18) of fractures occurred in the apical third of the root canal, while only four (19%) occurred in the middle third. The dimensions of the broken fragments ranged from 2 to 6 mm.

5044 root canals were instrumented as a result of the usage of 2,189 Premium Blue instrument files, all without fracture loss. Despite this, fractures did happen in 22 (1.03%) teeth and 22 (0.43%) root canals. The T25 and T20 files exhibited a particularly high fracture rate when taking into consideration the various sizes of Premium Blue instruments. With 11 (50%) and 10 (45.45%) separations, respectively, these files accounted for 21 (95%) of the breaks. There was one break in the T40 file and none in the T50 file.

With seven fractures in the upper molars and 11 in the lower molars, molars incurred the greatest number of fractures when analyzed by tooth type. On the other hand, the anterior teeth exhibited the lowest frequency of fractures, with just one fracture in the lower anterior teeth and none in the upper anterior teeth. In particular, the incidence of fractures was 0.00% in the case of upper anterior teeth, 0.47% in the case of lower anterior teeth, 0.69% in the case of upper premolars, 0.32% in the case of lower premolars, 1.36% in the case of upper molars, and 2.25% in the case of lower molars [26].

The findings from multiple research are consistent, which emphasizes the importance of following a single-use policy for reciprocating systems to reduce fracture rates. In addition, although accidental fractures can happen during the root canal preparation process the improvements in alloy composition and instrument design have helped to lower the risk of fracture.

Limitations

The findings in this study should be further investigated through prospective clinical randomized research due to the shortage of information available for cross-sectional retrospective investigations using databases.

## Conclusions

The research concluded that the average fracture incidence for teeth was modest, at 1.03% for teeth and 0.43% for root canals. The greatest incidences of fracture were identified in the molar teeth, namely, in the mesiobuccal canals of the mandibular and maxillary molars. The apical portion of the root canals was where most fractures happened, and file sizes T20 and T25 were most commonly impacted. A minimal prevalence of fracture was seen during the root canal preparation process when Premium Blue devices were used, demonstrating their dependability and efficacy in everyday use.
